# Multivessel Coronary Artery Disease in Cancer Patients Undergoing Percutaneous Coronary Intervention: A Systematic Review and Meta-Analysis

**DOI:** 10.3390/life15040571

**Published:** 2025-04-01

**Authors:** Konstantinos C. Siaravas, Michail I. Papafaklis, Amalia I. Moula, Lampros K. Michalis, Chrissa Sioka, Christos S. Katsouras

**Affiliations:** 1 First Cardiology Department, University Hospital of Ioannina, 45500 Ioannina, Greece; 2 Division of Cardiology, Faculty of Medicine, School of Health Sciences, University of Patras, 26504 Rio, Greece; 3 Achilopouleio General Hospital of Volos, 38222 Volos, Greece; 4 Second Cardiology Department, University Hospital of Ioannina, 45500 Ioannina, Greece; 5 Department of Nuclear Medicine, University Hospital of Ioannina, 45500 Ioannina, Greece

**Keywords:** coronary artery disease, cancer, multivessel coronary artery disease, percutaneous coronary interventions

## Abstract

Cancer patients have a higher propensity for adverse cardiovascular outcomes, primarily due to the toxic effects of chemotherapeutic agents and radiation therapy. The objective of this systematic review and meta-analysis was to investigate the proportion of multivessel coronary artery disease (MVD) in cancer compared to non-cancer patients undergoing percutaneous coronary intervention (PCI). We systematically screened the literature for studies providing data on MVD in patients with and without cancer who underwent PCI. Seventeen observational studies (5200 patients with active cancer/history of cancer and 55,146 control patients without cancer) were included in the analysis. Most studies did not show statistically significant differences in the incidence of MVD. Overall, there was no significant difference in MVD occurrence in the cancer group (risk ratio [RR]: 1.03; 95% confidence intervals [CI]: 0.99–1.08; *p* = 0.19). A high degree of heterogeneity was observed among the studies (I^2^ = 57.32%). Further sub-analysis using only the six studies with matched control populations did not show significant differences in MVD between those groups (RR; 0.99, 95% CI: 0.94–1.05, *p* = 0.79). In addition, a subgroup analysis with patients who had acute coronary syndrome, who received radiation treatment, and in studies with cancer patients with active cancer did not change the statistical results. Our report highlights that there was no significant difference in the incidence of MVD between patients with and without cancer. Further research is needed to clarify the detailed characteristics of coronary artery disease in cancer patients.

## 1. Introduction

Cardiovascular diseases and cancer are common comorbidities. They share many common risk factors and there are many common genetic and molecular mechanisms leading to this close relationship [1,2,3]. Many risk factors of coronary artery disease (CAD) can also raise the incidence of cancer. Increasing age, male gender, obesity, unhealthy diet, dyslipidemia, diabetes, tobacco use, and socioeconomic stress are among the most significant [2,3]. Inflammatory processes and an increase in inflammatory mediators are commonly found in both diseases, highlighting the common pathophysiologic mechanisms of inflammation in both diseases’ progression [1]. Increased inflammatory mediators such as IL-1 and high sensitivity CRP are found in cancer and CAD patients. All these induce cancer cells’ mechanisms of survival and also induce oxidative stress. In addition, in CAD, oxidative stress mediates LDLoxidation, lipid accumulation, and inflammatory cells’ migration in atherosclerotic plaque [1]. Chemotherapeutic agents used for cancer treatment have many adverse cardiovascular effects, they may cause hypertension, angina, and CAD [4,5,6,7,8,9,10], while new cancer treatments lead to greater risk of vascular toxicities [8]. In addition, CAD is an important complication of radiation therapy [9,10,11,12,13]. Because of the toxicity of several chemotherapeutic agents and of radiation therapy on coronary arteries, one can speculate that cancer patients may have greater incidence and complexity of CAD (i.e., multivessel vs. single-vessel disease) at the time of percutaneous coronary intervention (PCI).

However, to the best of our knowledge, no systematic reviews or meta-analyses have studied the extent of CAD in cancer populations compared to non-cancer patients. The integration of available research data could potentially provide insights into the distinct management and outcomes of patients with cancer [14,15,16].

There are data from meta-analyses that cancer patients have higher mortality compared to non-cancer patients [17]. The present systematic review and meta-analysis summarizes the literature data on the incidence of MVD in patients with cancer undergoing PCI compared to patients without cancer. The objective of the current analysis was to investigate the incidence of MVD in patients with cancer undergoing PCI compared to patients without cancer.

## 2. Materials and Methods

### 2.1. Search Strategy and Selection of Studies

This systematic review and meta-analysis was conducted in compliance with the updated Preferred Reporting Items for Systematic Reviews and Meta-Analyses (PRISMA) 2020 statement [18]. The current review was not registered to any systematic review registry. 

Only the PubMed database was searched until December 2023. The following search terms were used with no additional filters: (coronary artery disease OR acute coronary syndromes OR percutaneous coronary intervention OR acute myocardial infarction OR ST-segment elevation myocardial infarction (STEMI) OR Non-STEMI (NSTEMI) OR unstable angina) AND (cancer OR malignancy). The main search terms used were coronary artery disease and cancer and all the other searches were carried out in a combination of two keywords in pairs.

The following inclusion criteria were applied: (1) a randomized control trial or an observational study design; (2) English publication language; (3) studies including data about MVD, or separately for three-vessel and two-vessel coronary artery disease in cancer patients undergoing PCI compared to patients without cancer; and (4) acceptance of only papers with high quality assessment to reduce possible bias. The patient populations were defined as (1) cancer population including patients with active cancer or a history of cancer that underwent PCI and (2) control group including patients without cancer that underwent PCI.

The exclusion criteria for ineligibility of articles in the present review include the following: (1) case reports, editorials, and reviews; (2) non-English language papers; (3) studies without data about MVD (e.g., studies with data for number of lesions but not vessels); (4) studies without a non-cancer control group; (5) studies with cancer patients in the control group; (6) studies with patients who have not undergone PCI; and (7) studies with an intermediate or poor-quality assessment rating or inconsistent reported data.

### 2.2. Data Extraction and Quality Assessment

References and abstracts of search results were screened by one author (K.C.S.) for relevance to the meta-analysis topic. After the initial assessment, the remaining publications were further screened independently for the inclusion and exclusion criteria (K.C.S., A.I.M., and M.I.P.). Disagreements were solved by consensus or a third independent author (C.S.K., C.S., or L.K.M.). Study quality and risk of bias were assessed by an independent reviewer using the NIH quality assessment tool and the Newcastle Ottawa scale for observational studies (performed by K.C.S. and M.I.P.). One reviewer (K.C.S.) performed data extraction using a standard spreadsheet.

The NIH quality assessment scale uses a 14 criteria yes or no or cannot determine, not applicable, and not reported questions for the assessment of validity of observational cohort studies, resulting in categorizing studies in Good, Fair, or Poor ratings. The Newcastle Ottawa scale assesses risk of bias in the following 3 domains: selection of the study groups, comparability of groups, and ascertainment of exposures. Studies with scores of less than 4 were considered to have a high risk of bias, those with scores of 4 to 6 an intermediate risk of bias, and those with scores of 7 or more a low risk of bias [19]. In our analysis, in order to eliminate the risk of bias, we included only the papers with low risk of bias with an NIH quality assessment of good and an Ottawa score more than 7. 

### 2.3. Statistics and Data Analysis

The total numbers of patients diagnosed or not with MVD in each patient group (cancer vs. control) were extracted directly from the publications and used for the analyses. Similarly, data on the main cardiovascular risk factors were also directly extracted from the publications. The results are presented as risk ratio (RR) with 95% confidence intervals (CIs). Heterogeneity across studies was determined by the Cochran’s Q statistic, and I^2^ was also computed. Data from individual studies were combined using a randomeffects model with inverse variance weighting. Pooled estimates (i.e., % of cardiovascular risk factors) of the studies were obtained as a weighted average by fitting the randomeffects model. Funnel plots and Egger’s regression test were used to assess publication bias. Additionally, we checked whether a single study disproportionally influenced the pooled results by performing a sensitivity analysis using the leave-one-out method. Also subgroup analysis was conducted for patients with acute coronary syndromes, patients that have received radiation treatment, and for cancer populations that included patients with active cancer. Tests were two-tailed and a *p*-value < 0.05 was considered statistically significant. The statistical software package Stata 17.0 (Stata Corp LLC, College Station, TX, USA) was used for the analysis.

## 3. Results

### 3.1. Literature Search and Study Selection

**Figure 1:**
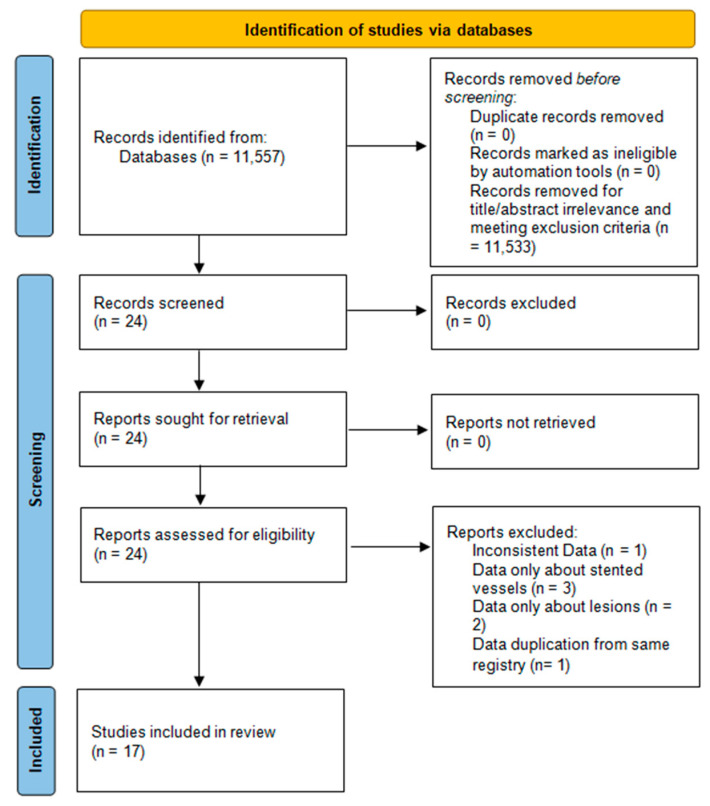
Flowdiagram of publication selection after systematic literature review.

We reviewed in total 11,557 publications and excluded all publications with irrelevant titles and abstracts. After exclusion of case reports, reviews, editorials, and duplicate publications, 24 publications were collected for further processing. Data availability and inclusion criteria were assessed, and quality assessment rating was used to categorize regarding risk of bias. In total, seven studies were excluded. One study was excluded because of inconsistent data in the published paper [20]. Three studies were excluded because they only presented information about the number of stented coronary vessels and did not present data about MVD [21,22,23]. Two studies were excluded because they provided data about coronary lesions and did not define if the lesions were on different vessels or on the same coronary vessel [24,25]. One study provided patients’ information from the same registry as another study (that was included in the analysis); thus, of the two studies, we selected the one with the larger patient sample in order to avoid duplication of the included patients [26]. After the exclusion of these 7 papers, the remaining 17 studies fulfilled all eligibility criteria. Figure 1 shows the flow diagram for the selection of papers.

### 3.2. Characteristics of Eligible Studies

Table 1 shows the 17 selected studies. The vast majority of these studies included patients with both acute coronary syndromes (ACSs) and chronic coronary syndromes (CCSs) [27,28,29,30,31,32,33]. No particular definition was given for MVD in the included papers and no functional indices (e.g., fractional flow reserve) were used.

Three studies included only patients with ST-segment elevation myocardial infarction (STEMI) [34,35,38] and five studies included patients with acute myocardial infarction, defined as either STEMI or non-STEMI [36,37,39,40,43]. No randomized controlled trials were conducted on cancer patients undergoing PCI. Fourteen studies had a retrospective observational design, while three employed prospective data collection [34,37,38]. 

The definition of cancer and the patient inclusion criteria were diverse in the studies. The analyzed papers included patients with active cancer or history of cancer in various organs and tissues. Eleven studies included patients with active cancer during PCI [27,28,29,30,33,34,36,38,39,40,41], five included patients with active cancer or any history of cancer [30,31,34,41,42], and one included only patients with historical cancer since active cancer status was an exclusion criterion [38]. Three studies included patients with radiation therapy before PCI [27,29,30]. While all studies included a control group, six studies used a matched control group of patients [27,29,30,31,35,42] and five out of six studies with a matched control group used propensity score matching [27,29,30,31,42].

### 3.3. Patient Characteristics

Overall, there were 5261 cancer patients and 54,879 non-cancer patients. Table 2 presents the demographics, risk factors, and previous myocardial infarction history in patients with and without cancer history from all the analyzed studies. Except for diabetes, there were statistically significant differences in all other risk factors for cardiovascular disease between patients with a history of cancer and the control patients. There was a higher incidence of hypertension (72.3% vs. 70.1%, *p*-value: 0.045) and previous MI (25.2% vs. 23.8%, *p*-value: 0.029) in the patient group with cancer, while there was a higher incidence of hyperlipidemia (63% vs. 58.8%, *p*-value: 0.007) and smoking (45.1% vs. 41.2%, *p*-value: 0.026) in the group without cancer. Thus, there are substantial differences in the incidence of risk factors for CAD between the two comparison groups that may influence the overall results of MVD.

### 3.4. Mortality Data

All the examined studies provided data on all-cause mortality rates either in-hospital or after various follow-up periods up to 6 years. All the studies that contained relevant data reported higher overall mortality and cardiac mortality rates in cancer patients compared to patients without cancer. We did not conduct a meta-analysis on cancer patient outcomes after PCI because they already exist [17]. We refer only to descriptive data about mortality from the included studies. 

In-hospital all-cause mortality, as reported by three studies [30,34,37], was higher in cancer patients. In addition, the mortality rates of cancer and non-cancer patients at 1 year, as reported by seven studies [27,34,36,38,40,41,42], and mortality at 5 years, as reported by seven studies [28,29,30,31,32,37,39], were higher in cancer patients compared with the control patients.

In-hospital cardiovascular mortality was reported by only two studies [34,37], 1 year cardiovascular mortality rates were reported by four studies [34,37,40,41], and five year cardiac mortality rates were reported in five studies [28,30,32,37,39] and were also higher in cancer patients compared with control patients. 

According to radiation treatment exposure, Liang et al. showed a reduced overall survival (cardiac and cancer related) in cancer patients that received radiation treatment [27] and Reed et al. reported a higher all-cause and cardiovascular disease mortality in cancer patients [29]. Landes et al. reported a higher cardiac death and a higher rate of new myocardial infarction and target vessel revascularization in cancer patients [31]. Nakatsuma et al. presented a higher non-cardiac (cancer related) mortality but also higher cardiac mortality in cancer patients. Furthermore, they showed higher heart failure rehospitalization rates and an increase in non-cardiac surgery procedures [32]. Velders et al. reported a higher all-cause mortality, higher cancer-related mortality in patients with cancer diagnosis of <6 months, and higher cardiovascular mortality of cancer patients [34]. Wang and Nozaka et al. presented a higher non-cardiac mortality in cancer patients [35,39]. Finally, Tanimura et al. reported a higher major adverse cardiovascular events rate and new coronary ischemic events in cancer patients [43]. 

### 3.5. Extent and Complexity of CAD

Only two papers [29,42] contained information about the syntax score of the patients. Reed et al. reported that patients undergoing external beam radiation therapy had a syntax score of 9 (6–15) (median, IQR range) and controls without cancer had a score of 10 (6–15), respectively (*p* = 0.270) [29]. Mrotzek et al. also reported comparable syntax scores between patients with cancer 4 (0–12) and without cancer 6 (0–13; *p*-value: 0.391) [42]. One study that was included in this review used intravascular optical coherence tomography (OCT) imaging data for further pathophysiologic plaque characterization [43]. A higher prevalence of plaque erosion (60.3% vs. 36.5%, *p*: <0.001) and calcified nodule (22.2% vs. 12.6%, *p*: <0.001), whereas a lower prevalence of plaque rupture (17.5% vs. 50.9%, *p* < 0.001), were observed on culprit lesions of cancer patients with acute coronary syndromes compared with the control patients.

### 3.6. Incidence of Multivessel CAD

**Figure 2:**
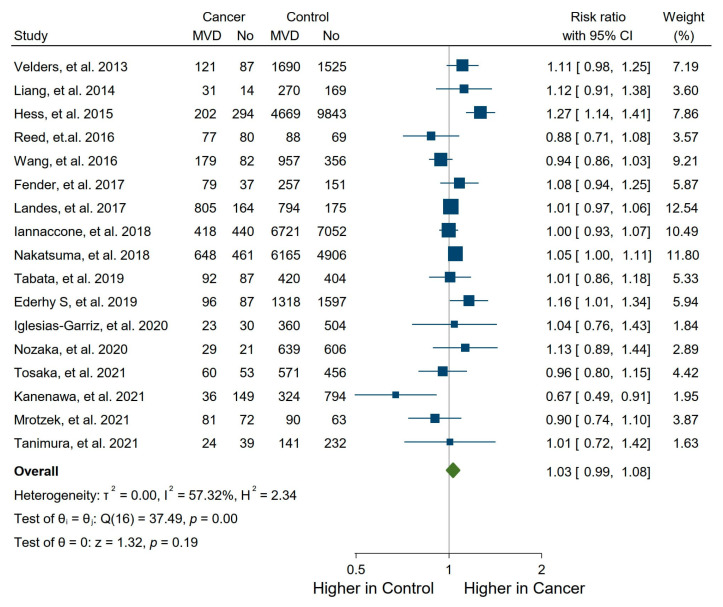
Forest plot of all studies [27,28,29,30,31,32,33,34,35,36,37,38,39,40,41,42,43] regarding impact of cancer on frequency of multivessel disease (MVD) using random effects model.

Out of a total of 17 studies, there were 2 studies showing a clearly significantly higher incidence of MVD in the cancer patient group with RRs of 1.27 (95% CI: 1.14, 1.41) and 1.16 (95% CI: 1.01, 1.34) (Figure 2) [28,37]. Only one study reported a significantly higher incidence of MVD in control patients without cancer (RR: 0.67; 95% CI: 0.49, 0.91) [40]. In the remaining 14 studies, there were no statistically significant differences in the frequency of MVD. In 11 of these 17 studies, the frequency of MVD was numerically higher (RR ≥ 1.01) in cancer patients, whereas only in 5 studies, the frequency of MVD was numerically higher in the control group (RR ≤ 0.99), and in 1 study, there was no difference between the groups (RR: 1.00). 

**Figure 3:**
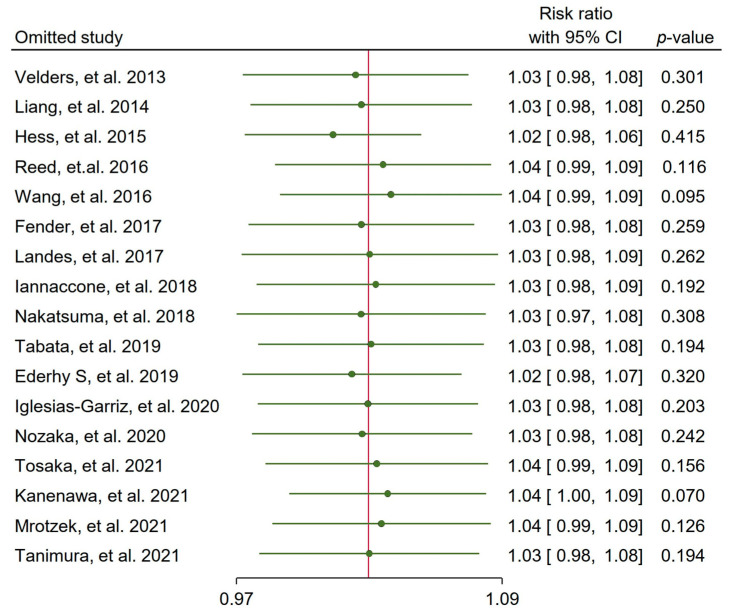
Leave-one-out sensitivity analysis with risk ratios (confidence intervals) for multivessel disease using random effects model [27,28,29,30,31,32,33,34,35,36,37,38,39,40,41,42,43].

Overall, the meta-analysis did not show a statistically significant higher incidence of MVD in the cancer group of patients (RR: 1.03, 95% CI 0.99–1.08, *p* = 0.19; Figure 2). However, there was a high degree of heterogeneity among the studies (I^2^ = 57.32%). A Funnel plot including all studies did not show significant publication bias (Egger’s test, *p*-value 0.314; Supplemental Figure S1). The sensitivity analysis using the leave-one-out method showed that no study could significantly change the statistical significance of the overall result (Figure 3).

**Figure 4:**
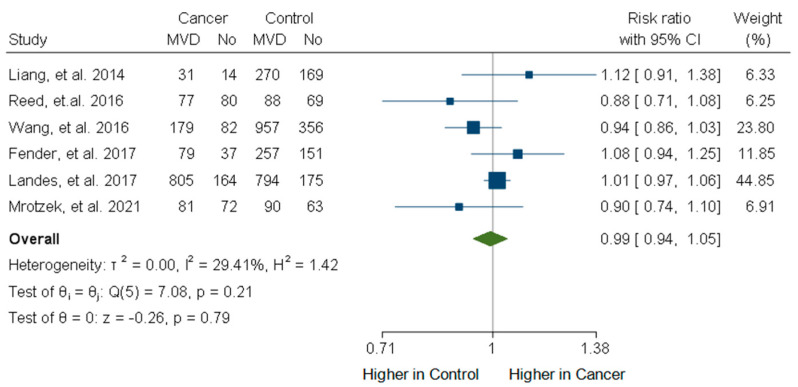
Forest plot of studies [27,29,30,31,35,42] with matched control patient group regarding impact of cancer on frequency of multivessel disease (MVD) using random effects model.

Taking into account the observational nature of the included studies, as part of our sensitivity analyses, we separately analyzed the studies which included a matched control group in order to reduce possible bias due to confounding variables. Overall, the six studies with a matched control group did not show a significant difference between the cancer and the matched control patients (RR: 0.99, 95% CI: 0.94–1.05, *p* = 0.79; Figure 4). The heterogeneity in this subgroup of studies was moderate (I^2^ = 29.41%). The Funnel plot including this subgroup of studies again did not show significant publication bias (Egger’s test *p*-value of 0.77; Supplemental Figure S2). Further subgroup analysis was performed for patients with acute coronary syndromes, radiation treatment, and active cancer status. The forest plot for patients with acute coronary syndromes, reduced heterogeneity I^2^: 26.87%, but did not show a significant difference in MVD between cancer and non-cancer patients with an RR:1.02, CI: [0.97, 1.09] (Supplemental Figure S3). In addition, subgroup analysis of patients that received radiation treatment did not show a statistically significant result with an RR: 1.03, CI: [0.90, 1.18] and a heterogeneity of I^2^: 39.77% (Supplemental Figure S4). Finally, subgroup analysis for studies that included patients with active cancer showed non-significant differences in the results with RR: 1.02, CI: [0.92, 1.14] and a high level of heterogeneity with I^2^: 74.5% (Supplemental Figure S5). 

## 4. Discussion

The present meta-analysis did not show a statistically significant higher incidence of MVD in cancer compared to non-cancer patients. Our findings could not support the initial hypothesis of higher expected incidence of MVD in cancer patients, but no definitive conclusion can be drawn mainly due to the aforementioned differences in study populations and the high level of heterogeneity.

Statistically significant differences in baseline characteristics between the cancer and control patient groups were observed in most of the selected studies. More specifically, there were significant differences in the percentages of risk factors for CAD between comparison groups, especially in studies that were not using a matching methodology [36,38]. Only six studies used a matched control group in order to limit confounding bias; however, heterogeneity remained moderate in our sensitivity analysis with only these studies. Furthermore, different proportions of patients that underwent PCI for ACS or CCS were observed between studies, so that some studies contain more patients with ACS while others contain more patients with CCS as an indication for PCI. In our study, in the cancer patient group, there was a higher frequency of hypertension and history of previous MI, whereas in the control group, there was a higher frequency of hyperlipidemia and smoking. These statistically significant differences in risk factors between the groups of patients may contribute to the absence of a significant difference in MVD between the comparison groups. Ideally, one would like to have access to the raw data of all studies and thus perform patient-level meta-analysis to adjust for confounders such as hypertension, hyperlipidemia, and smoking.

Patients with cancer have been excluded from large randomized trials; thus, there is a lack of evidence regarding the incidence of MVD in that setting. Current comparative data on MVD in patients with cancer are mainly based on retrospective and prospective observational studies; this issue is reflected by the high degree of heterogeneity we observed in our analysis. No randomized control studies are currently present and there are limited prospective observational studies [34,37,38]. 

Another factor contributing to the heterogeneity is that the types of cancer and the cancer status of target groups among studies varied. Some studies included patients with active cancer, defined as recently diagnosed cancer, recurrent or progressive disease, or currently receiving treatments, while other studies accepted patients with a history of cancer without further defining their status. Different proportions of cancer patients with active versus a past history of cancer are noted among the studies. Due to the retrospective design of most studies, there is limited information on the chemotherapeutic agents of cancer patients or on previous exposure to radiation treatment. 

The current meta-analysis focuses on MVD in cancer patients compared with patients without cancer. Previous meta-analyses in cancer patient populations focused on outcomes and mortality between groups [44,45]. Worse outcomes and increased mortality rates have been reported in cancer patients [17]. However, based on our results, this cannot be attributed to the presence of MVD. On the other hand, there are many well-known vascular toxicities of cancer chemotherapeutic treatments leading to CAD [4], while increased inflammation and endothelial injury of radiotherapies impose a great risk [44,46]. It is possible that the retrospective design of most studies and the differences in the selected patient populations cause underestimations of MVD rates in cancer patients. Another explanation could be the higher rates of non-ruptured plaque mechanisms of CAD and lower percentage stenosis in cancer patients, which may explain the non-significant rates of angiographically MVD between cancer patients and controls [43]. Tanimura K. et al. reported that plaque erosion was more often a mechanism of acute coronary syndrome in culprit lesions of patients with cancer than in those without. Moreover, most available studies did not provide details on the number of lesions, which may also reflect the extent of CAD, but they only reported the number of vessels with CAD [43]. 

Two of the included studies used SYNTAX score to provide more information on the complexity of CAD but without any significant differences between the two patient groups [29,42]. We speculate that patients with radiation therapy exposure may have lower SYNTAX scores because patients with previous radiation exposure may have only ostial or proximal lesions rather than diffuse disease including bifurcation or trifurcation lesions. In addition, we included only studies with patients who underwent PCI, while many more could have undergone catheterization only for coronary angiography, which may have been revascularized with CABG or may even have been managed with conservative treatment.

Cancer treatments such as chemotherapy and radiation treatment impose further cardiotoxic effects on the heart and increase the incidence of CAD. There are many cardiotoxic effects of chemotherapy such as heart failure, cardiomyopathies, arrhythmias, CAD, pericardial disease, pulmonary embolism, and venous thromboembolic disease [47]. Many pathogenetic mechanisms have been proposed for CAD in patients treated with chemotherapy. Chemotherapeutic drugs impose endothelial dysfunction and impair vasorelaxant mechanisms (impaired nitrous oxygen production), leading to enhanced endothelial permeability and increased vascular tone. Inflammatory cells secrete cytokines and migrate in the tunica media of the vessels. Those mechanisms induce the early stages of atherosclerosis. Furthermore, chemotherapy impairs vascular reparability properties, increases oxidative stress with reactive oxygen species production, and induces pro-inflammatory pathways. All of these increase atherosclerotic plaque formation and make plaques more vulnerable [48]. 

Radiation treatment can lead to endothelial cell injury and microvascular dysfunction. Radiation increases reactive oxygen species and increases the oxidative stress of vascular cells. In addition, radiation impairs DNA repair through DNA strands breaking. It also enhances enzymatic phosphorylation, protein misfolding, and degradation impairing molecular cellular mechanisms. Furthermore, radiation increases many inflammatory markers such as tumor necrosis factor, interleukin-6, platelet-derived growth factor, and transforming growth factor, leading to atherosclerosis formation [49]. These mechanisms of chemotherapy and radiation therapy increase the incidence of CAD, something that was not confirmed with statistical significance for multivessel CAD in our analysis. 

Another common issue on the treatment of cancer patients is the usage of antiplatelet agents and the management of the antithrombotic treatment. Cancer patients were excluded from the clinical trials of patients with PCI for CAD, since cancer patients may have contraindications for the use of antiplatelet drugs or they are at increased risk of complications. In the current review, the most common second antiplatelet agent on the papers used in the analysis was clopidogrel in combination with aspirin. Seven of the studies used in the analysis report no significant differences on dual antiplatelet agents used between cancer and non-cancer patients [26,34,36,37,40,41,43]. Liang et al. showed a significantly lower use of clopidogrel in cancer patients [27] and Mrotzek et al. showed a lower use of aspirin in cancer patients [42]. On the other hand, three papers showed a significantly higher use of clopidogrel in cancer patients [29,32,38].

There are difficulties in the management of patients with CAD and cancer. Especially challenging is the management of patients with active cancer. First of all, cancer patients that are receiving chemotherapy may have variations in blood pressure or have drug interactions of their cancer treatment with medications used for CAD risk factor management, leading to defective primary or secondary CAD prevention. Cancer increases patient thrombotic and hemorrhagic risk simultaneously, leading to difficulties in the management of proper antithrombotic treatment. Patients may have severe myelosuppresion during chemotherapy, leading to low hemoglobin levels and platelet counts [50]. A shorter dual antiplatelet drug duration may be appropriate for patients with very high bleeding risk. In addition, multidisciplinary decisions and consensus are needed between several medical specialties for patients with CAD and newly diagnosed cancer, especially for those patients that are planning on having an operation. Best decisions about the revascularization procedures are important because of the need for dual antiplatelet treatment and its duration, which may delay operation on the cancer [51]. Furthermore, in patients with cancer and MVD, revascularization with CABG may be an option. CABG may impose some risks for patients that are undergoing thoracic irradiation because of delayed wound healing or reduced patency of the grafts. Finally, many cancer patients have a metastatic disease, have a short life expectancy due to their disease stage, have many comorbidities, and those who are frail may not be candidates for a revascularization treatment and their CAD may be managed only medically or by having only palliative treatment [50].

Future research should focus on conducting well-organized prospective studies. Additionally, future prospective studies should focus on (1) detailed data about two- versus three-vessel disease or left main disease, especially after radiation therapy; (2) data on intravascular imaging from cancer patients; and (3) functional assessment of lesions in patients with cancer history. All the above will provide a more complete understanding regarding the extent of CAD in cancer patients. 

The limitations of our meta-analysis mainly reflect the observational nature of all studies included. Data about chemotherapeutic agents or previous radiation therapy were not available, thereby restricting important associations between MVD and specific therapies. Differences in population selection about baseline characteristics and significant differences in CAD risk factors, indications for PCI, and cancer status or type of cancer may have influenced the overall results. Furthermore, since our meta-analysis is not a patient level analysis, there is a limitation on conducting further subgroup analysis on patients with cancer according to cancer type, cancer stage, cancer status, and in patients with chronic coronary syndromes. Study populations included mixed solid cancer and hematologic malignancy populations and in nine studies, a mixed ACS and CCS population was used, while there were no exact data on how many patients had MVD. Lastly, there is a possibility of selection bias because we included only papers with cancer patients that underwent PCI, while there may be patients with MVD who underwent coronary artery bypass grafting (even though active cancer status might be a cause for being turned down for cardiac surgery). We excluded patients with CAD that underwent CABG and patients that were treated with conservative treatment, which is another limitation of our analysis. 

## 5. Conclusions

Our analysis did not show any statistically significant difference in MVD incidence between cancer patients and non-cancer patients with CAD undergoing PCI, but no definite conclusions can be made. Our report highlights the heterogeneity and limitations of available data regarding the incidence of multivessel CAD in cancer compared to control patients. Even after further analysis using the six studies with a matched control population and subgroup analysis of patients with ACS, radiation exposure, or with active cancer, there were no statistically significant differences in MVD between the comparison groups.

## Figures and Tables

**Table 1:** Summary of studies including details about patient groups and outcomes.

Study	PCI Cause	Exposed Group	Control Group	Primary Outcomes	Secondary Outcomes
Velders M. et al. [34]	STEMI	Active and history of cancer	Non-cancer	1-year all-cause mortality, cardiac mortality	1-year survival
Liang J. et al. [27]	ACS and CCS	EBRT before or after PCI	No EBRT history	TLR	MI, cardiac mortality, overall mortality
Hess C. et al. [28]	ACS and CCS	Recent and non-recent cancer	No pre-PCI cancer	Cardiovascular mortality	Composite cardiovascular mortality, MI, repeat revascularization, all-cause mortality
Reed G. et al. [29]	ACS and CCS	EBRT before PCI	Non-cancer	All-cause mortality	Cardiovascular mortality
Wang F. et al. [35]	STEMI	Cancer history	Non-cancer	In-hospital and long-term mortality	
Fender E. et al. [30]	ACS and CCS	EBRT	No EBRT history	All-cause mortality	Cardiac, non-cardiac mortality, procedural complications, angiographic characteristics, number of diseased vessels
Landes U. et al. [31]	ACS and CCS	Cancer history	Non-cancer	All-cause mortality, nonfatal MI, composite of death, TVR, CABG	Cardiac, malignant, infectious cause of death and other cause of death
Iannaccone M. et al. [36]	ACS	Active or <2 years cancer history	Non-cancer	1-year death or MI, bleedings	Death, re-infraction, bleeding after 1-year
Nakatsuma K. et al. [32]	ACS and CCS	Cancer history	Non-cancer	All-cause death, cardiac or non-cardiac death, HF hospitalization, stent thrombosis, TLR, stroke	
Tabata N. et al. [33]	ACS and CCS	Current or cancer history	Non-malignancy	1-year cardiovascular death, non-fatal MI, stroke, revascularizations	
Ederhy S. et al. [37]	STEMI and NSTEMI	Any cancer history	Non-cancer	5-year all-cause, cardiovascular, non-cardiovascular mortality	In-hospital complications (recurrent MI, VF, bleeding, transfusion, stroke)
Iglesias-Garriz I. et al. [38]	STEMI	History of cancer, no active cancer	Non-cancer	Totalmortality during follow-up	
Nozaka M. et al. [39]	AMI	Current and history of cancer	Non-malignancy	All-cause mortality, readmission for DHF	
Tosaka K. [40]	AMI	Active or history of cancer	Non-cancer	Cardiac death	Bleedings, non-cardiac death, MI, stroke
Kanenawa K. et al. [41]	CAD	Active or history of cancer	Non-cancer	1-year NACCE (all-cause death, MI, stroke, major bleeding)	Bleeding, thrombotic composite of MI, stent thrombosis
Mrotzek S. et al. [42]	ACS and CCS	Cancer history	Non-cancer	1-year all-cause mortality	5-year all-cause mortality
Tanimura K. et al. [43]	ACS	Active cancer and history of cancer	Non-cancer	Cardiac death (due to MI, arrhythmia, heart failure, sudden)	Non-fatal MI, revascularization of coronary vessel, stroke TIA, heart failure admission

ACS: acute coronary syndrome; AMI: acute myocardial infarction; CCS: chronic coronary syndrome; CS: Cardiogenic Shock; CAD: coronary artery disease (ACS and CCS); CAG: coronary angiography; DHF: Decompensate Heart Failure; EBRT: external beam radiation therapy; MI: myocardial infarction; NSTEMI: non-STelevation MI; PCI: percutaneous coronary intervention; STEMI: ST-elevation MI; TIA: Transient Ischeamic Attack; TLR: Target Lesion Revascularization.

**Table 2:** Demographics, risk factors, and previous disease history in patients with and without cancer from all analyzed studies.

	CANCER (N = 5261)	NO CANCER (N = 54,879)	*p* Value
Diabetes	35.9%	33.6%	0.068
Hypertension	72.3%	70.1%	0.045
Hyperlipidemia	58.8%	63%	0.007
Smoking	41.2%	45.1%	0.026
Previous MI	25.2%	23.8%	0.029

Percentages (%) are pooled estimates of the studies included and are obtained as a weighted average by fitting the random effects model. Data were available in 16 out of 17 studies for diabetes and hypertension (N = 4292 and N = 53,910, respectively); 14 out of 17 studies for hyperlipidemia (N = 2687 and N = 28,327, respectively); 16 out of 17 studies for smoking (N = 4402 and N = 41,106, respectively); and 16 out of 17 studies for previous MI (N = 4292 and N = 53,910, respectively). MI: myocardial infarction.

## Supplementary Materials

The following supporting information can be downloaded at: https://www.mdpi.com/article/10.3390/life15040571/s1, Supplemental Figure S1: Funnel plot including all studies did not show significant publication bias (Egger’s test: *p*-value = 0.314); Supplemental Figure S2: Funnel plot including only studies with matched control group for assessing publication bias (Egger’s test: *p*-value = 0.77). Supplemental Figure S3: Forest plot of the studies including only patients with an acute coronary syndrome: impact of cancer on the frequency of multivessel disease (MVD) using a random effects model. Supplemental Figure S4: Forest plot of the studies including only cancer patients with radiation therapy: impact of cancer on the frequency of multivessel disease (MVD) using a random effects model.

## Abbreviations

The following abbreviations are used in this manuscript:

ACS	Acute coronary syndrome
CABG	Coronary artery bypass grafting
CAD	Coronary artery disease
CCS	Chronic coronary syndrome
CI	Confidence interval
MI	Myocardial infarction
MVD	Multivessel disease
NSTEMI	Non-ST elevation myocardial infarction
OCT	Optical coherence tomography
PCI	Percutaneous coronary intervention
RR	Risk ratio
STEMI	ST elevation myocardial infarction
