# Supplementary material for: Multivessel Coronary Artery Disease in Cancer Patients Undergoing Percutaneous Coronary Intervention: A Systematic Review and Meta-Analysis
Source: Life (Basel). 2025 Apr 1;15(4):571. doi: 10.3390/life15040571 (PMC12029082; doi:10.3390/life15040571)

SUPPLEMENTARY MATERIALS

Multivessel coronary artery disease in cancer patients undergoing percutaneous coronary intervention: A systematic review and meta-analysis

Konstantinos C. Siaravas [1], Michail I. Papafaklis [2] , Amalia I. Moula[3] , Lampros K. Michalis[4], Chrissa Sioka[5,*] and Christos S. Katsouras[1]

Supplemental Figure S1. Funnel plot including all studies did not show significant publication bias (Egger's test: p-value=0.314).

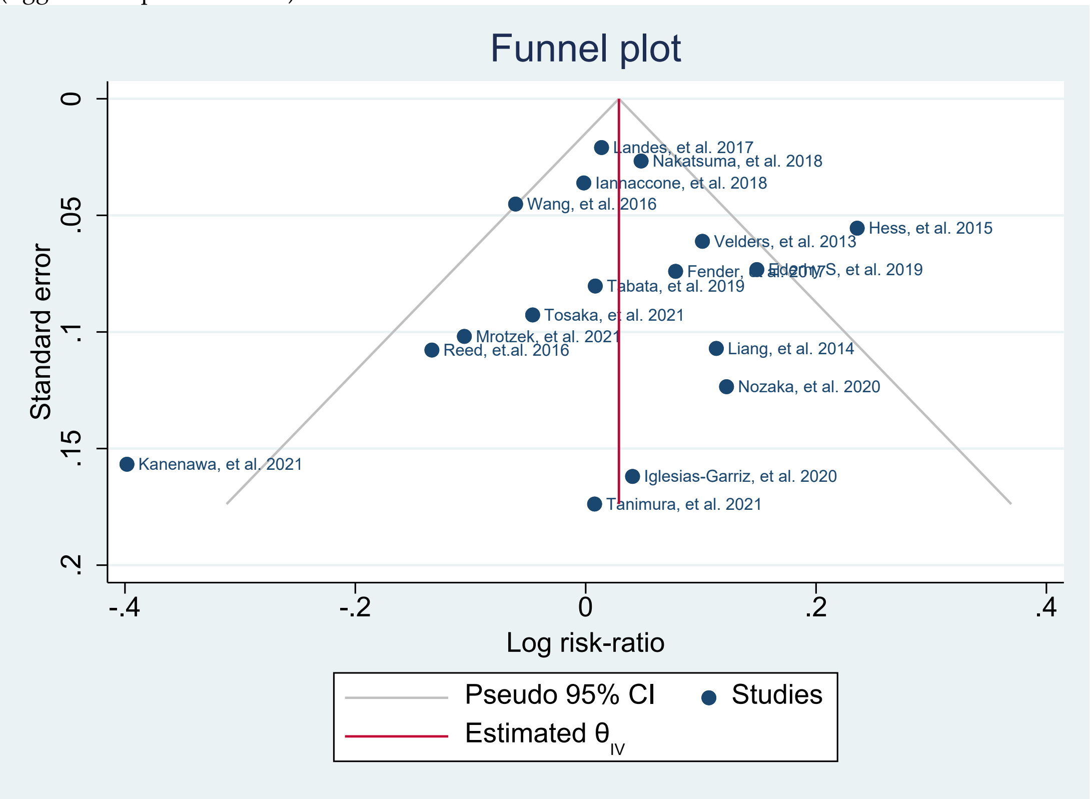

Supplemental Figure S2. Funnel plot including only the studies with a matched control group for assessing publication bias (Egger’s test: p-value=0.77).

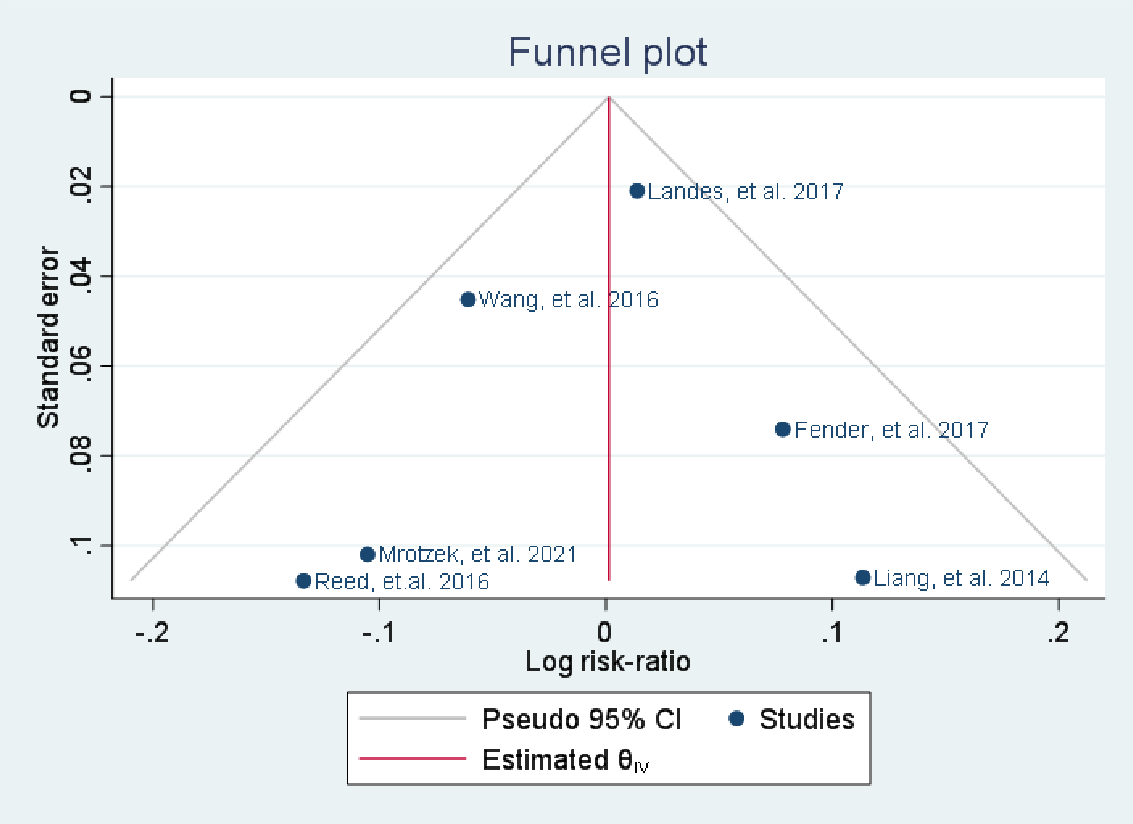

Supplemental Figure S3. Forest plot of the studies including only patients with an acute coronary syndrome: impact of cancer on the frequency of multivessel disease (MVD) using a random effects model.

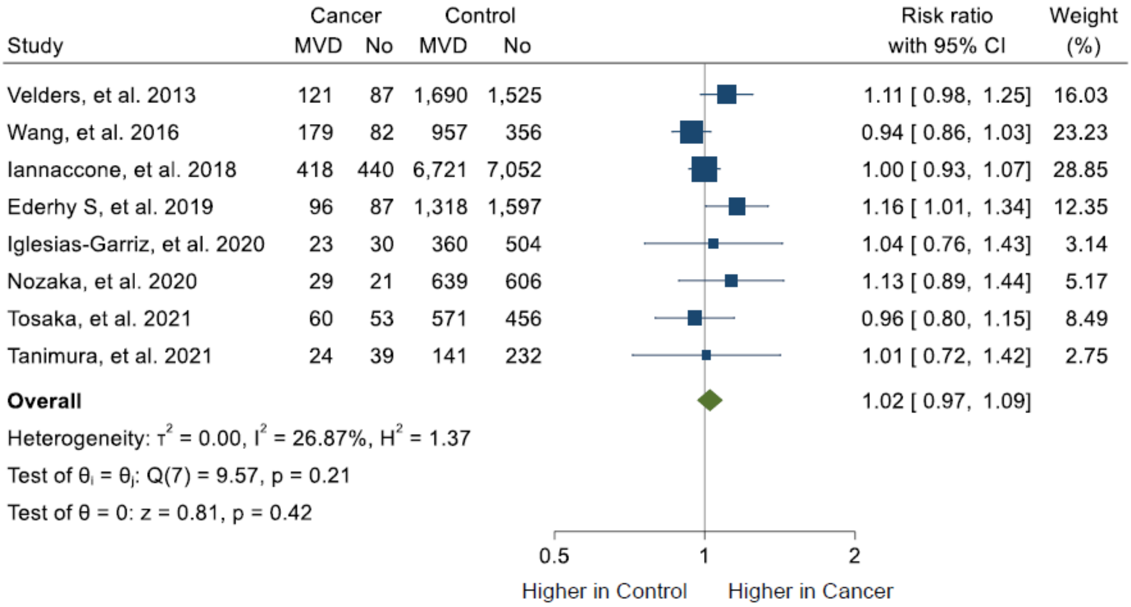

Supplemental Figure S4. Forest plot of the studies including only cancer patients with radiation therapy: impact of cancer on the frequency of multivessel disease (MVD) using a random effects model.

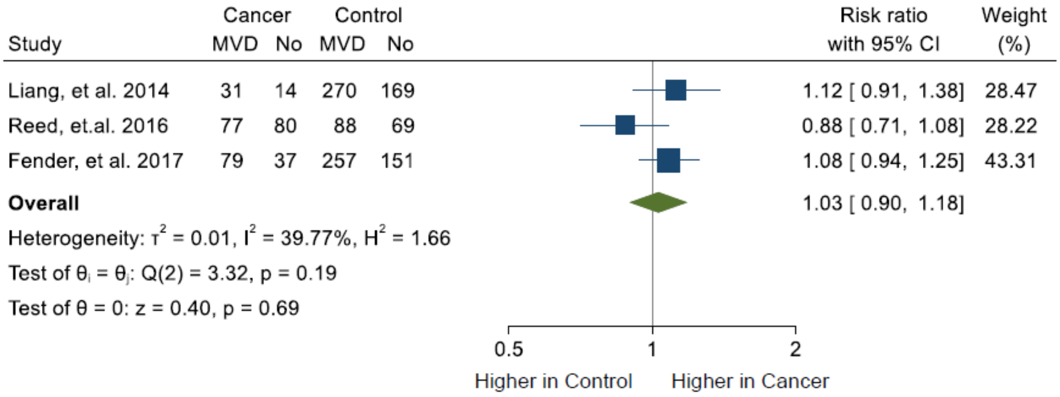

Supplemental Figure S5. Forest plot of the studies including only patients with active cancer: impact of cancer on the frequency of multivessel disease (MVD) using a random effects model.

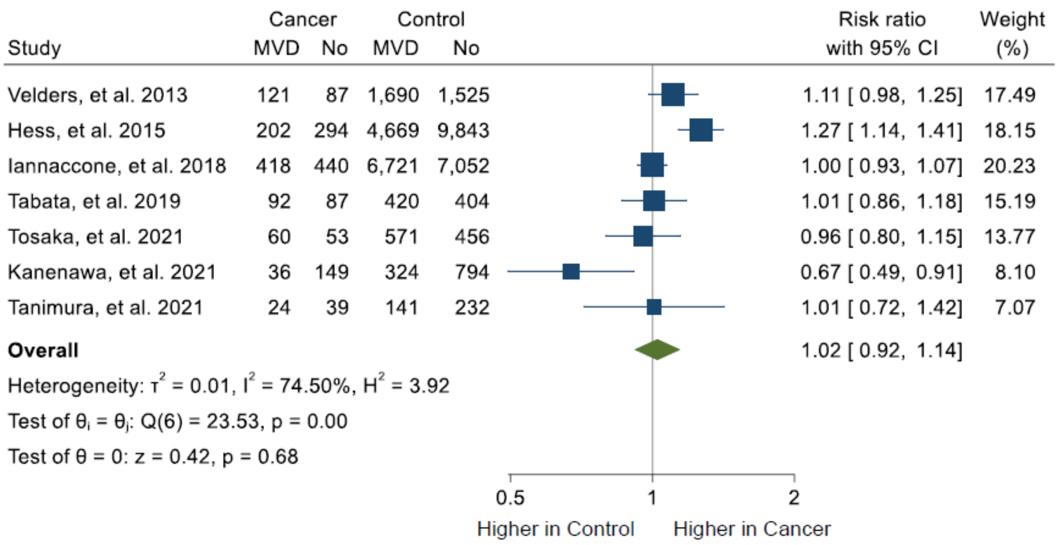

Supplement: Supplementary file 1 [file life-15-00571-s001.zip › life-3522759-supplementary.pdf]
